# Promoting the Learning of Basic Sciences in a Changing Small-Group Learning Landscape. Interviews of tutors and students in a medical program

**DOI:** 10.15694/mep.2018.000008.1

**Published:** 2018-01-09

**Authors:** Jane Gair, Graeme Horton, Sarah Wojcik, Alixandra Wong

**Affiliations:** 1 University of Victoria/ University of British Columbia; 2 University of Newcastle; 3 Dalhousie University; 4 University of Victoria

**Keywords:** Problem-based learning, faculty development, basic science learning

## Abstract

This article was migrated. The article was marked as recommended.

Tomorrow’s medical graduate needs to be equipped today to be able to make sense of continued advancements in medical science and then apply these to the benefit of patents and communities. It is important that current methods of teaching and learning in medical programs such as problem-based learning are subject to critical evaluation as to whether they are fit for purpose, and many medical programs are questioning whether problem-based learning provides adequate grounding in basic science. In this context, we interviewed twelve tutors and four students of one distributed medical program in order to explore their views on what elements of PBL promoted understanding of basic science. Our participants reported that setting a clear agenda about basic science learning was crucial; that tutor preparation and guides needed to take account of different tutor backgrounds; particular styles of prompting and questioning from the tutor were valuable; and that students could benefit from being primed with key introductory concepts about how basic sciences related to each other prior to commencing PBL. These findings will be useful for medical curriculum developers who are seeking to innovate with their curriculum but wish to retain what may be currently most valuable by stakeholders.

## Introduction

Medical curricula must constantly evolve in the face of changing population demographics, health priorities, and advances in the basic and clinical sciences. Those involved in the process of curriculum renewal strive to improve methods of learning in ways that address emergent challenges. One challenge facing medical programs is to ensure that medical students learn sufficient foundational basic sciences amongst the expanding plethora of topic areas which all need to be covered in an integrated curriculum.

With medical education constantly being re-imagined and re-designed, curriculum developers are continuously searching for new models to improve how students learn and develop a suitable knowledge base that matches the current innovations in medicine ( Mahan & Clinchot, 2014). Problem-based learning (PBL) has been widely implemented as an innovative pedagogical approach that has become the predominant teaching and learning strategy in medical programs ( Samarji, 2014).

Though the current literature is replete with articles describing the many benefits of problem-based learning in medical education, there is also much published on the potential disadvantages of PBL ( Hmelo-Silver, 2004; Gulpinar *et al.*, 2015; Zhang, 2015). Ribeiro (2011) points out the higher-class unpredictability of PBL and increased time and workload for the tutor. Lim warns that PBL, which is augmented by conventional teaching methods, so-called “hybrid PBL” will be dysfunctional and inferior to the traditional approach from which the PBL emerged (Lim, 2012).

There has been much discussion as to whether PBL is more effective at delivering basic science knowledge than traditional teaching methods. Previous studies have evaluated the effectiveness of PBL versus non-PBL methods in teaching basic science knowledge by testing students’ academic abilities using performance-based measures ( Hmelo-Silver, 2004). Many researchers suggest no significant difference exists when evaluating knowledge levels between these curricula ( Prince *et al*., 2003; Samarji, 2014).

In the eyes of some, PBL, which was once revolutionary, is now passé. A number of medical programs are now moving away from PBL towards other methods of small-group learning, some of which allow for greater delivery of content during tutorials. These developments can be seen as opportunities for innovation in which new learning technologies can be combined with use of successful elements of current or past practice.

PBL is recognized as an engaging method of integrated learning. Al-Shaikh *et al.* (2015) examined second year medical students’ perceptions of PBL and found that 90.4% of the 52 students responding to the survey agreed that PBL integrates basic science with clinical knowledge. This was higher than their agreement with other study questions about their perceptions of PBL; that it stimulates critical thinking (82.7%), stimulates self-learning (73.1%), enhances problem-solving skill (65.4), and identifies knowledge gaps (59.6%) ( Al-Shaikh *et al.,* 2015).

However, there is little known about learners’ and facilitators’ perceptions of what has specifically motivated, challenged, and facilitated medical students’ learning of the basic sciences within the context of a PBL environment. The purpose of this small study was to explore the perceptions of some students and tutors about what they considered to facilitate learning of the basic sciences in PBL tutorials in one medical program; what specific techniques and group processes which they valued the most. This may be relevant to medical programs whether currently using PBL, considering moving towards PBL, or critically examining alternative methods of small group learning as part of curriculum renewal.

## Methods

In order to explore the experiences of students and tutors in the participants’ PBL environments and to enable fresh insights to emerge, we used semi-structured interviews and qualitative analysis using a Modified Grounded Theory Approach. This study took place at the University of British Columbia (UBC) MD Undergraduate Program in Vancouver, British Columbia and in Victoria, British Columbia at the University of Victoria, two of the four distributed sites of the provincial program.

### Ethics Approval

Approval of the ethics committees was obtained from the universities of the participants (IRB from UBC: H12-02766).

### Recruitment

A convenience sample of both tutors and students of two sites of this distributed medical program was recruited, in that all PBL tutors and students who were on site and available were invited to participate. There were four students who participated and twelve PBL tutors who were interviewed: all were interviewed during that week except one tutor who was interviewed by Skype a week later.

### Conducting of Interviews

There were two pairs of medical students and two pairs of tutors who were each interviewed for one hour. The other eight tutors were interviewed individually for between 30 and 60 minutes. Data were collected through digital audio recording of interviews. Each interview was conducted by the same investigator, and was transcribed verbatim.

### Analysis of Interviews

Inductive analysis by three of the investigators evoked categories for the responses and these were converted to 42 codes. Data analysis was an iterative process whereby the investigators separately considered how the responses related to the codes and then came together for comparison of their conclusions. The process of questioning the meanings we inferred from the data served to clarify the definition of codes, and to delineate the relationships between categories of responses. Involving more than one analyst is said to be valuable when researcher bias may be perceived to be a problem ( Pope *et al.*, 2000) and to help ensure that we were open to new possibilities in the data. This allowed for clarification and challenging of any assumptions we might have held prior to interviewing our subjects.

## Results

### Participants

Two students were in their first year and two were in their second year of an undergraduate medical program. Of the tutors interviewed, eight had a basic science background and four had a clinical background. Three of the tutors had current Faculty Development roles and one had a previous senior Faculty position. Two tutors had current responsibility for parts of the curriculum. Two students and six tutors were from one site of the distributed program, and the rest were all from one other of the sites. The interviews are numbered 1-12 and indicated “tutors” or “students” according to who the interview was conducted with.

### Findings

Optimal and effective learning of the basic sciences was often equated with deep learning, which involved students being challenged and using their own initiative in considering and understanding physiological and pathological mechanisms that underlie patient presentations.

*I was one of these people that never in a million years would I like this stuff -good old fashioned didactic learning you know; sit in the lecture and go to sleep. And then problem based came on and I was sucked in, because it is good. [.] because it does give them the opportunity to delve deep. (9 Tutor)*

Engagement between learners and the written patient case, their tutors and each other was considered a requisite for deep learning and for understanding to be retained.

*In Problem-Based Learning there has to be a problem that they can relate to, that they feel is useful for them to understand the underlying basic science behind it, not just :“this is an, another academic exercise I’ve gotta do.” (11 Tutor)*

Deep learning and engagement were seen as dependent on factors relating to each of the students, to the tutor, and to the design of the PBL case. The means by which engagement of learners was best promoted could be grouped according to the following categories:

**1. Management of students wrestling with key concepts.** It was stated by a number of tutors that students learn best when they are in the active role of solving problems for themselves. This can however require a sustained effort and tolerance of uncertainty at times on the part of the student and the tutor.

*If it’s handed out to them on a platter there’s not so much..interaction in the PBL room and there’s not so much of them actually coming to wrestle with the problem (2 Tutor)*

The students discussed that they had varying degrees of tolerance of this type of process:

*I find it very difficult to apply some of those concepts to more complex problems like you would see in a patient, especially when I don’t really have a very strong background in those things. And so it’s like, sometimes I feel like I’m just barely. like treading water and just, with all of this, trying to just learn all of that, the little, little details and all the small parts and trying to apply it to the big concepts.(6 Students)*

How the learning is paced throughout the session, and how the tutorials and rest of the students’ week is structured can help determine students’ application to the solving of challenging problems. Provision of content by experts during lectures can usefully support the learning which occurs during the tutorials, but if this is not well aligned or designed to complement the tutorials, can reduce student engagement.

*Making sure that the interplay between the PBL tutorials and the lectures is really well structured, and that the lectures build off of the case and vice versa. As opposed to covering the same material, which has happened in some of our weeks and it can be really frustrating for the students and for the tutors when the students are sitting there saying, “Well, we don’t want to discuss this. We know we have a lecture on this topic in an hour.” (10 Tutors)*

There were suggestions that students may best be primed with certain concepts and relational understanding of different sciences prior to commencing a PBL curriculum. This was especially thought to be the case for students who had limited or no previous learning of basic science prior to their medical studies

*There may well be a place for an introductory course of some kind with maybe some kind of PBL in it and some non-PBL in it, to bring people up to speed. So that when they hit the, the, whatever mechanism you’re going to learn the basic sciences in, at least have some language, and they at least have some concepts. (1 Tutorss)*

There were a number of types of questions which tutors could ask which were considered valuable to prompt learning.

*When we were discussing something and we were going to move on to something else and he would go “Have you considered” something, something, something. And he wouldn’t say exactly what it was but he would kind of you know, hintingly ask something that would generate a bit of thinking amongst the group and then we would be discussing things and we would kind of hit on whatever important topics. (6 Students)*

One tutor succinctly stated that the best questions were those which students ask of each other. Examples of specific types of questions asked by tutors that were thought to be valuable by our participants are listed in Table 1.

**2. Professional development and preparation of tutors.** Deep learning and student engagement was in part dependent on effective professional development of tutors. It was understood that tutors have accountability to optimise opportunities in which students can exercise their responsibility to learn.

*And you make sure that you go through the manual before you go in there, and you think about how you want to direct them to reaching the directives before you actually get into that room so you’re always thinking: we need to get to this objective, or this learning issue by the end of this two hour session. (1 Tutors)*

*My job is not to tell them; my job is to make sure that they figure it out. (9 Tutor)*

In order to do this, tutors need to be diligent in their preparation and use of tutor guides.

*If you have a tutor who’s not comfortable with this given basic science concept, they’re not gonna push too hard because, and if they do, they’re not gonna know.. And so the tutor guides are really absolutely crucial for that. If the tutor guide’s written properly, in theory any non-expert should be able to handle that session with the students and get to the learning objectives. (12 Tutor)*

**3. Fostering of a supportive learning environment in touch with its learners.** It was suggested that it can be important for the group members and the tutor to be aware of what the students’ experience is with the tutorial’s content area at the start of a tutorial. However, provided there is a kind and accepting attitude within the group, this could emerge during the tutorial discussion and allow students to build on each other’s learning.

*It’s like a phenomenal process when you get a room of people together and you all think that you don’t know anything about it..And then somebody says something minor and you’re like “Oh, I do know that, I remember reading something about that a long time ago” and “Isn’t that how this is connected?” And two hours flies by in that, in that environment and you really feel confident once you leave. (6 Students)*

Tutors also discussed their enjoyment of spending time with enthusiastic students as they facilitated the students’ learning and learnt themselves from the discussion.

*You can see those students thinking and then at some point “Oh, they’ve got it!” Those beautiful moments, that’s the reason why I do this. (1 Tutors)*

**4. Rational use of resources.** How students are directed to resources and how learning from resources is then handled during the session needs to be considered and discussed amongst the group. There were seen to be problems with having too little restriction on students being able to access resources during the tutorial

*When we had fully open books, people would just print off pages from like Wikipedia or something and they would come and they would read to us and it was terrible. (6 Students)*

And there were also issues at times with too much restriction and rules around the use of resources.

*We also had to memorise a lot of primary sources and the years that they were released and stuff like that, so..you spend all your time memorising the Journal of Birth Defects and Gastroenterology 2002, you know, when you should be memorising the important parts, like the details, like the enzyme names. (6 Students)*

**5. Clarity of the agenda of learning basic sciences within small group learning.** If learning of basic science is to be a focus of the learning process then this must be made clear to students.

*This is not: “how do we diagnose and treat this patient and move on with our lives?” but the exercise is for them to say “what basic information do we need to understand what’s going on with this patient?” As long as they’re aware of that I think it’s effective. (11 Tutor)*

*Making sure you have the type of PBL that you want for your university defined. And everybody on the same page, I think that’s really necessary as well. (6 Students)*

It was interesting that many of the tutors commented on the students preferring to pass over the basic sciences in order to concentrate on clinical content,

*..they’re not as interested in learning the basic science.. they’re now getting closer and closer and closer to becoming doctors on the ward or mini doctors on the ward. And so they kind of want to gallop towards the clinical scenario, rather than go through the process of the in between. (1 Tutors)*

Whereas the students interviewed felt that time in PBL was in fact best devoted to learning of the basic sciences.

*For me, those are the most valuable aspects of PBL, is when we just step back and go through the general, normal physiology or anatomy. (8 Students)*

**6. Orientation of tutors and students to the place of small group tutorials within students’ learning program.** It was seen as important for tutors and students to understand how the learning in each tutorial fits with the rest of the curriculum. It was also seen as valuable to indicate to students how the learning fits with their later professional lives. Students may best be specifically cued to understand how basic science will help them to solve future problems.

*When we’re talking in a small group and the family doctor throws in like, “This is actually a really important concept because you will be asked about this on the wards.” Or, “Your patients are gonna want this explained.” ..I like when I’m learning biochemistry that seems so distant from the clinic to be told why this is relevant. (8 Students)*

Having well-constructed PBL cases with an appropriate balance of basic science and clinical learning was seen as necessary for students to achieve knowledge and skills transferable to the wide range of fields in which graduates of a medical program may practice, including clinical and research areas.

*From a learner’s perspective, they said that if, they could sense it when the case is not written properly to incorporate that basic science concept in the proper context or in the real scenario,. that’s when it can turn them off. (10 Tutors)*

*The case, the way it’s written will either direct you to more basic science or more clinical. It’s the way they set the case to be. (1 Tutors)*

Making it clear to all tutors how every session relates to an overall curriculum map was considered important but aspirational, and a potential valuable inclusion to each tutor guide.

*I think if we were able in our tutor guides to explicitly say: “This is the question for the students. In this block of this year, they learned this, this was the learning objective, this was the level of depth, this is how this should build on that previous knowledge.” I think that would go such a long way to improving how those concepts are learned and built on through the progression of the students through PBL. (10 Tutors)*

## Discussion

Problem-Based Learning made its debut in 1969 at the medical school at McMaster University and since then has been introduced in virtually every medical school in some form. Whilst the efficacy of PBL has been debated, researched, analyzed, critiqued, and discussed in the literature for many years with much variability, PBL can be described as a very successful innovation ( Prideaux, 2007), given its widespread adoption in medical education around the world. It has been said that PBL is particularly successful in the early years of medical training where it is an effective vehicle for covering the basic sciences ( Prideaux, 2007).

“Wear and tear” of long-term PBL programs has been described ( Moust *et al*. 2005) where a focus on content rather than process can erode the benefits of active learning at the core of PBL. With comprehensive faculty development and continued renewing and reviewing of the curriculum, this can be addressed ( Young & Papinczak, 2013). As well, a consistent definition of PBL along with a guide to the practices that encompass it are as elusive and varied as the number of schools who have adopted some version of it ( Taylor & Miflin, 2008). That PBL is “a continuum of approaches rather than one immutable process” and “a teaching method that can be included in the teacher’s tool-kit along with other teaching methods rather than used as the sole educational strategy” (Davis & Harden, 1999) remains true now in 2017. Neville (2008) suggested that the foundational principles of PBL are found in all curricula that define themselves as PBL, whether or not they adhere to the original strict definition put forth by Barrows ( 1986, 2000). Therefore, a PBL approach to learning embodies the flexibility and adaptability necessary to accommodate the demands for new curricular outcomes and graduating competencies as they arise.

Medical school curriculum developers must navigate a way through these tensions, and be diligent in assessing the efficacy and appropriateness of the pedagogies and methods used for teaching and learning. It is important to continually look at new strategies and to select teaching and learning methods so that they facilitate the match between content and outcomes ( Prideaux, 2007).

In this limited exploration of PBL, we obtained perspectives of members of two stakeholder groups involved in one medical school curriculum: the medical students and the PBL tutors; with particular focus on the learning of basic sciences in the PBL setting and classroom. To our participants, of critical importance was the engagement of learners with each other, with the PBL cases, and with their PBL tutors. Learners, tutors, and faculty can promote this by being clear about the purpose of PBL, and placing sufficient emphasis on the goal of learning basic science.

In order to mitigate the “wear and tear” there needs to be a continual emphasis on facilitation skills and faculty development of PBL tutors. Young and Papinczak (2012) describe a process whereby each term tutors attend short mandatory faculty development workshops in order to re-establish the goals and practices of the PBL process in order to decrease bad habits. The workshops could include specific questioning techniques and other specific skills that can reduce dissonance between the student expectations of what PBL is, and what behaviours the tutor is demonstrating. Tutor guides are also an important feature of preparing the tutor, but the tutor guide will mean different things to different people depending on whose hands they are in. For example, a tutor with a basic science background in biochemistry will have no trouble with a case about glucose metabolism, whereas someone with a sociology background may struggle with a basic science content heavy case. This may not be one-size-fits-all, and the use of supporting materials to provide extra foundational understanding may also need to be available or referenced in the guides.

Another important aspect of preventing divergence of the curriculum and the learning experience and intended outcomes in PBL, is the need for students to be prepared and kept up to speed with the agenda. Our participants also proposed a priming course or a set of lectures, but again, as with tutor guides, this may not be one-size-fits-all either. With the shift away from prerequisites for medical school, the student body will have varied backgrounds and experiences when it comes to the basic sciences and different students may need to be supported in different ways and we suggest that future research into this could be useful.

### Limitations

The authors acknowledge that the small sample size of our study is a limitation and would not be indicative of the full range of perceptions of students or tutors at this institution.

## Conclusion

Curriculum developers must constantly re-evaluate how teaching and learning methods are serving the goals of the program. In order to support small group learning of basic science utilizing clinical cases there are benefits to keeping learners in an active role, in providing sufficient guidance and support for tutors, and in promoting engagement and supportive learning environments.

It is essential that there is clarity of the agenda of small group learning and that everyone is on the same page. If learning basic science is part of the agenda, it needs to be obvious to everyone - to students, tutors, and curriculum developers. All stakeholders should be regularly consulted so that everyone is part of the conversation about the best teaching and learning methods, including PBL and determining the best ways to support the diverse and rich mix of students and tutors who enjoy and benefit from the process.

## Take Home Messages

• Innovation in medical programs often involves scrutiny of how well basic sciences are learned within problme-based learning.
• The aspects of problem-based learning which are most valued in promoting understanding of basic science can guide new models of small-group learning.
• Deep basic science learning is more likely to occur if it is a clear and explicit part of the agenda for students and tutors.
• Students can be specifically cued to understand how basic science understanding within each case scenario will help them to fulfil their future professional roles.
• Tutors may be more likely to facilitate sufficient time for basic science discussion if they are clear about where each tutorial’s learning sits within the whole curriculum.

## Notes On Contributors

Jane Gair is a Teaching Professor in the Island Medical Program, a distributed site of the University of British Columbia medical program in British Columbia, Canada. She is the Island Medical Program site lead for small group learning.

Graeme Horton is a Senior Lecturer in Medical Education and General Practice at the University of Newcastle, Australia, and is Program Convenor and Chair of the Teaching and Learning Committee of the Joint Medical Program of the University of Newcastle and the University of New England.

Alixandra Wong just completed her undergraduate degree at the University of Victoria in Victoria, British Columbia.

Sarah Wojcik is a graduate student at Dalhousie University in Halifax, Nova Scotia.

## Appendices

**Table 1.** Examples of specific questions asked by tutors in PBL that are valuable to the learners.

**“The right questions”: examples of useful questions cited by participants** *Questions about sources of information* • Where did you get that information? *Questions seeking clarification about mechanism* • What is the reason, what’s the mechanism, how come? • Why is it called that, what is the underlying mechanism that this describes? • Mary is cold but she is not shivering. Why is she not shivering? *Questions which probe reasoning* • Why is that, could you explain your reasoning? • What do you need to know before you do that? *Questions about consequences of processes* • Now knowing this about the body, what else do you think could happen? • What happens if this enzyme wasn’t working properly, what would be the downstream effects of that? *Questions linking to clinical application* • How does that fit with what the patient is experiencing? • How does this relate back to your patient? • How would you describe this to your patient? • But at what times? Is it constant or is it after meals? *Questions relating to directing the learning process* • Does somebody want to write that up on the board?
