# Supplementary material for: Promoting the Learning of Basic Sciences in a Changing Small-Group Learning Landscape. Interviews of tutors and students in a medical program
Source: MedEdPublish (2016). 2018 Jan 9;7:8. [Version 1] doi: 10.15694/mep.2018.000008.1 (PMC10711955; doi:10.15694/mep.2018.000008.1)
Supplement: Peer review history

# Reviewer report: Reviewer response for version 1

**DOI:** 10.21956/mep.19474.r28741

**Published:** 2018-05-30

**Authors:** Sateesh Babu Arja

**Affiliations:** 1 Avalon University School of Medicine

This review has been migrated. The reviewer awarded 4 stars out of 5 It is very well written paper with clear objectives and goals. I enjoyed reading this paper. There was a good discussion about the literature regarding. There are many papers citing the advantages of PBL Vs traditional curriculum. Equally, we have the same amount of research articles citing that there are no added advanatges. Many medical programs implemented the PBL curriculum for the last 20 to 30 years. Many medical schools out of them are going back towards other models of curriculum or implementing hybrid curriculum (combination of lectures and PBL sessions). There was a great deal of discussion about the hybrid model in the article by Hurt, M.M. and Harris, J.O. (2005) Founding a new College of Medicine at Florida State University. Academic Medicine, 8, 11, 973-979.The history of PBL in medical education goes back to the article by Shmidt in 1983. I was involved in implementing the PBL curriculum at our university. We don't use PBL as a whole curriculum but we are using PBL as a part of learning strategy. PBL sessions are scheduled in the weekly schedules. Our experiences correlate with the experiences cited by Al-Shaikh et al. (2015). The main goal of PBL for us is the integration of clinical sciences into the basic sciences. It also promotes critical thinking and self-directed learning.As authors mentioned in this paper, there is a limitation due to less number of participants. To conduct effective PBL, it needs to be well directed and faculty development activities are one of the key factors to success. Would like to see more papers in this area. This paper is interesting for faculty members, PBL tutors, curriculum developers, and academic administrators.

Reviewer Expertise:

NA

No decision status is available

# Reviewer report: Reviewer response for version 1

**DOI:** 10.21956/mep.19474.r28740

**Published:** 2018-04-09

**Authors:** Alex Noakes

**Affiliations:** 1 University of Liverpool

This review has been migrated. The reviewer awarded 3 stars out of 5 The introduction for this paper clearly set out why this study was needed in the context ofthe current medical education system. It is nice to see qualitative studies beingperformed and the study allows us to see individual thoughts on the PBL system,compared to quantitate results that PBL is commonly judged by. This paper is appropriatefor professionals working in a medical education curriculum role and it is still worthreading if you have an interest in medical education. The discussion provides usefulimprovements such as tutor guides and tutors specific to the session outcomes have beenidentified from the comments in the results. The paper does highlight concerns thattutors and students have about PBL, but also the positives they have taken from it too.The disadvantages of the paper are that the paper doesn’t focus too much on basicsciences and is quite broad during the discussion and interview process. Despite this, thetake home messages have been written to apply the results to basic sciences in themedical curriculum, this is helpful to take away and makes the paper easy to implement.A negative is that the interview sample is small, especially from students. The studentsthat provided interviews are in first and second year and therefore there is no samplefrom older years, this may have assisted with a more retrospective view. It could also besaid that PBL is the teaching style and that the real issue may lie with the set up of thecurriculum in regards to course content and focus, which may be the issue instead.Now that these themes have been brought out during this paper, it would be helpful toreproduce similar interviews at more medical schools and with more tutors and studentsto see if these themes are universal. (Student perspective)

Reviewer Expertise:

NA

No decision status is available

# Reviewer report: Reviewer response for version 1

**DOI:** 10.21956/mep.19474.r28739

**Published:** 2018-01-14

**Authors:** P Ravi Shankar

**Affiliations:** 1 American International Medical University

This review has been migrated. The reviewer awarded 4 stars out of 5 Problem-based learning (PBL) has become modified and varied as the authors rightly mention. In many schools it is used as a learning method rather than as a complete curricular strategy. The strengths and weaknesses of PBL have been discussed in the literature and some authors have raised concerns that a pure PBL curriculum may lead to deficiencies in knowledge of the basic sciences. This is an interesting and well-written manuscript. I was involved with a PBL program at an offshore Caribbean medical school, where PBL was being used a learning strategy while lectures continued to be the predominant method of instruction. In today’s world resources, especially electronic ones are easily available to students and this can impact the PBL process. The process of searching for information has become nearly instantaneous. We also faced the problem of repetition of information between lectures and the PBL sessions as mentioned by the authors. Students may not devote much attention and energy to a PBL session if they are aware that the material and concepts will be addressed shortly in a lecture. Also students are more likely to be interested in the clinical aspects of the case and in diagnosing and treating the patient. The sample size is small and this has been mentioned in the limitations. The paper provides an interesting perspective from a Canadian medical school about the learning of basic sciences in an educational program using PBL There are a few spelling errors which can be corrected.

Reviewer Expertise:

NA

No decision status is available

# Reviewer report: Reviewer response for version 1

**DOI:** 10.21956/mep.19474.r28736

**Published:** 2018-01-12

**Authors:** Subha Ramani

**Affiliations:** 1 Harvard Medical School, Brigham and Women's Hospital

This review has been migrated. The reviewer awarded 3 stars out of 5 The authors have conducted an appropriate qualitative study to explore student and tutor perspectives on whether basic science concepts were effectively introduced in PBL sessions. The paper is well written and I like the themes that emerge.However, the methods section is very brief, I am not sure what the authors define as convenience sampling and there is no mention of how they ensured trustworthiness of the study. I actually believe that quantitative methodology is not necessary to lend credibility to the findings. However, I have been given recommendations in the past about writing more details under methods to convince readers that the study has been undertaken with rigor. Finally, I am not sure these themes would be exclusive to PBL, they may well apply to many other forms of interactive learning.Educators who design or teach PBL sessions are likely to find this article useful.

Reviewer Expertise:

NA

No decision status is available

# Reviewer report: Reviewer response for version 1

**DOI:** 10.21956/mep.19474.r28738

**Published:** 2018-01-12

**Authors:** Maurizio Vertemati

**Affiliations:** 1 Dipartimento di Scienze Biomediche e Cliniche "L. Sacco"

This review has been migrated. The reviewer awarded 3 stars out of 5 The manuscript illustrates an interesting approach to evaluate the relationship between PBL and the learning of basic science. It underlines that a deeper critical view of basic science concepts is mandatory to motivate the students and to improve their performance prior to commencing PBL, by stimulating an active learning process in close co-operation with the tutors. In this contest, a curriculum map in the PBL Tutor guide is very interesting and it could be a specific added value to approach medical curriculum. In my opinion, the only criticism is that this study would have been much more convincing if the conclusions were substantiated through quantitative assessments on a large number of participants, so to confirm the results and the impact of your study on students' knowledge acquisition.

Reviewer Expertise:

NA

No decision status is available

# Reviewer report: Reviewer response for version 1

**DOI:** 10.21956/mep.19474.r28737

**Published:** 2018-01-11

**Authors:** Gerard Flaherty

**Affiliations:** 1 National University of Ireland Galway

This review has been migrated. The reviewer awarded 4 stars out of 5 I enjoyed reviewing this paper. The authors provide a clear rationale for their study and frame this well against the existing literature on problem-based learning. While the central focus on how PBL promotes an understanding of the basic sciences gets diluted later in the manuscript, some very interesting insights have been captured from the semi-structured interviews. There were particularly rich data regarding how PBL might be optimised to facilitate the transition to learning of key concepts by students. I also appreciated the focus on embedding PBl sessions in a clinical context so that students grasped their future clinical relevance. The suggestion to incorporate a curriculum map in the PBL Tutor guide is surely to be encouraged. Table 1 is a very helpful resource for less experienced PBL facilitators.

Reviewer Expertise:

NA

No decision status is available

# Reviewer report: Reviewer response for version 1

**DOI:** 10.21956/mep.19474.r28735

**Published:** 2018-01-09

**Authors:** Trevor Gibbs

**Affiliations:** 1 AMEE

This review has been migrated. The reviewer awarded 3 stars out of 5 After I read this paper I was at a little bit of a loss in understanding what the main message was from the author's research. Like my co-reviewer I thought that more could be made available from a larger sample of both students and tutors, although I feel that an attempt was made to take a cross sample from two distributed centres. Many of the answers raised to the questions were interesting and could form a basis of discussion in themselves, but to me , one element that I felt I was not shared with was the way that PBL had been introduced in the school(s) and specifically what training in PBL methodology both students and tutors had prior the adoption of PBL as a learning strategy. There seemed to be a discrepancy between PBL as a teaching tool as opposed to its real purpose of being a facilitation tool to promote student learning and directed self learning. I did feel that Table 1 was a useful take away lesson for those new to small group activities and how to ask the right questions.

Reviewer Expertise:

NA

No decision status is available

# Reviewer report: Reviewer response for version 1

**DOI:** 10.21956/mep.19474.r28734

**Published:** 2018-01-09

**Authors:** Mohammed Osman

**Affiliations:** 1 General orgainization for teaching hospitals

This review has been migrated. The reviewer awarded 3 stars out of 5 An interesting manuscript.Positive points; -It has a clear objective which was (to explore the experiences of students and tutors in the participants’ PBL environments and to enable fresh insights to emerge). -Ethical approval was included- It was based on an assessment of the opinion of both students and tutors. Negative points;-Small number of participants-Most participants were from one site- Students in their first years- It was based on qualitative assessment. Probably adding some statistical association would be beneficial to improve the power of the studyOverall, I beleive the study is interesting to readers. However, I m not sure weather these results can be generalised based on a small number of participants. It needs more conformatory trials.

Reviewer Expertise:

NA

No decision status is available
